# Diverse Effects of ANXA7 and p53 on LNCaP Prostate Cancer Cells Are Associated with Regulation of SGK1 Transcription and Phosphorylation of the SGK1 Target FOXO3A

**DOI:** 10.1155/2014/193635

**Published:** 2014-04-22

**Authors:** Meera Srivastava, Ximena Leighton, Joshua Starr, Ofer Eidelman, Harvey B. Pollard

**Affiliations:** Department of Anatomy, Physiology and Genetics and Institute for Molecular Medicine, Uniformed Services University of Health Sciences (USUHS), School of Medicine, Bethesda, MD 20814, USA

## Abstract

Tumor suppressor function of the calcium/phospholipid-binding Annexin-A7 (ANXA7) has been shown in *Anxa7*-deficient mice and validated in human cancers. In the androgen-resistant prostate cancer cells, ANXA7 and p53 showed similar cytotoxicity levels. However, in the androgen-sensitive LNCaP, ANXA7 greatly exceeded the p53-induced cytotoxicity. We hypothesized that the p53 underperformance in LNCaP could be due to the involvement of p53-responsive SGK1 and FOXO3A. In this study, we show that p53 failed to match programmed cell death (PCD) and G1-arrest that were induced by ANXA7 in LNCaP. WT-ANXA7 preserved total FOXO3A expression with no hyperphosphorylation that could enable FOXO3A nuclear translocation and proapoptotic transcription. In contrast, in the p53-transfected LNCaP cells with maintained cell proliferation, the phosphorylated (but not total) FOXO3A fraction was increased implying a predominantly cytoplasmic localization and, subsequently, a lack of FOXO3A proapoptotic transcription. In addition, p53 reduced the expression of aberrant SGK1 protein form in LNCaP. Using Ingenuity Pathway Analysis and p53-signature genes, we elucidated the role of distinct SGK1/FOXO3A-associated regulation in p53 versus ANXA7 responses and proposed that aberrant SGK1 could affect reciprocal SGK1-FOXO3A-Akt regulation. Thus, the failure of the cell growth regulator p53 versus the phospholipid-binding ANXA7 could be potentially attributed to its diverse effects on SGK1-FOXO3A-Akt pathway in the PTEN-deficient LNCaP.

## 1. Introduction

The tumor suppressor gene (TSG) function of the Ca/phospholipid-binding Annexin-A7 (ANXA7, NP001147.1, NCBI) has been demonstrated in our previous studies, including* Anxa7(+/−)* murine model and ANXA7 tissue microarray profiling in normal versus tumor tissues [1, 2]. Loss of heterozygosity at the ANXA7-harboring 10q-locus [3] and a particular loss of ANXA7 expression in the hormone-refractory prostate tumors provided evidence for the hormone-related tumor suppressor role of ANXA7 in prostate cancer. ANXA7 matched cytotoxicity of a conventional tumor suppressor p53 in androgen-resistant DU145 and PC3, but greatly surpassed p53 effects in androgen-sensitive LNCaP [4]. We also showed that ANXA7 protected normal prostate cells and induced RB-associated cytotoxicity in prostate cancer cells* in vitro* [4]. The RB-associated ANXA7 effects in LNCaP included a reversal of the RB-dependent repression of the proapoptotic E2F-transcription. We undertook this study to understand the molecular mechanisms that caused p53 underperformance compared to ANXA7 and, subsequently, to elicit the beneficial tumor suppressor mechanisms of ANXA7 in LNCaP.

The PTEN-mutant LNCaP possess a constitutively active Akt which negatively regulates the forkhead transcription factor FOXO3A/FKHRL1. A FOXO3A decrease followed by the p53-downstream p27 promoter transactivation was reported in the LNCaP progression to androgen independence [5]. In a crosstalk between two transcription factors, p53 and FOXO3A [6, 7], the activated FOXO3A impaired p53 transcriptional activity, while the activated p53 inhibited the FOXO3A-mediated transcription via FOXO3A phosphorylation and cytoplasmic retention. While a major FOXO3A-regulator Akt was not essential for the p53-dependent FOXO3A suppression, the serum/glucocorticoid regulated kinase 1, SGK1, was significantly induced in a p53-dependent manner. SGK1 is a target of p53 which can repress the glucocorticoid receptor (GR) transactivation and binding to SGK1-promoter [8]. The androgen-sensitive and PTEN-mutant LNCaP lack the SGK1-inducing GR [9] and TGF-beta [10, 11]. Consequently, the p53-responsive prosurvival SGK1, which facilitates androgen receptor- (AR-) dependent [12] and FOXO3A-mediated [13] cell survival, could be specifically involved in the p53 underperformance in LNCaP.

Hence, we studied the SGK1/FOXO3A-associated effects of p53 versus ANXA7 that were anticipated to reveal the alterations in canonical p53 cell survival control as well as beneficial ANXA7 tumor suppressor effects in the PTEN-deficient LNCaP.

## 2. Material and Methods

### 2.1. Cell Culturing and Infection

Normal prostate (PrEC) and androgen-sensitive LNCaP prostate cancer cells (ATCC) were cultured as suggested by the manufacturer and transfected with ANXA7 and p53 constructs. Adenoviral vector-based plasmids for infection (AdEasy System, Johns Hopkins Oncology Center) are “empty” vector, wild-type (WT) or dominant-negative (DN) ANXA7, and p53. WT-ANXA7 corresponded to a more abundant short ANXA7-isoform (NP 001147.1, NCBI). DN-ANXA7 (which is known to inhibit WT-ANXA7-induced phosphatidylserine liposome aggregation) contained triple mutations against calcium-binding site which was intended to affect COOH-terminal residues in annexin repeats 2, 3, and 4 (E277 → Q277, D360-E361 → N360-Q361, and D435-D436 → N435-N436, resp.).

### 2.2. Programmed Cell Death (PCD) and Cell Cycling Analysis

Programmed cell death (PCD) detection was performed in single GFP-positive cells. Early (phosphatidylserine exposure) and late (membrane permeabilization) PCD stages were studied by Annexin V-PE Apoptosis Detection Kit I (BD Pharmingen) and flow cytometry (EPICs XL-MCL, Beckman Coulter). DNA fragmentation was studied in the end-stage PCD by TUNEL- (terminal deoxynucleotidyl transferase dUTP nick end labeling-) based APO-BRDU Kit (BD Pharmingen) and flow cytometry (LSRII, BD Biosciences). To avoid the overlap with GFP-marker, FITC-labeled anti-BRDU mAb was substituted with the PE-conjugated anti-BRDU mAb (BD Pharmingen). Results were analyzed as the mean ± SE, and compared using an independent two-sample *t*-test at *P* < 0.05 level of significance. Cell cycle analysis was performed in parental and transfected (vector-, WT/DN-ANXA7-, or p53-) LNCaP cells (18 h), using propidium iodide staining (Sigma-Aldrich) and flow cytometry (ModFit LT, Verity Software House and EPICs XL-MCL, Beckman Coulter).

### 2.3. Western Blotting

Western blotting was performed using standard procedures, and equal amounts of total protein were electrophoresed on 4–20% Tris-Glycine gels and MagicMark (Invitrogen, Carlsbad, CA, USA). Antibodies used are FOXO3A and phospho-FOXO3A-Ser318/321 (numbers 9467 and 9465, resp., Cell Signaling Technology); SGK1 (number 3272, Cell Signaling Technology) and *β*-actin were used as control.

### 2.4. RNA Extraction and PCR

Confluent parental and vector-, WT/DN-ANXA7-, and p53-transfected LNCaP and DU145 cells were harvested, and total RNA was isolated with RNAqueous-4 PCR Kit (Ambion, Austin, TX, USA) and used for reverse transcription (SuperScript First-Strand Synthesis System for RT-PCR, Invitrogen, Carlsbad, CA, USA). SGK1 C-terminal fragments were amplified in duplex PCR using the following primers: SGK1 (forward—5′-CTCCTGCAGAAGGACAGGA-3′; reverse—5′-GGACAGGCTCTTCGGTAAACT-3′) and beta-actin (forward—5′ CTGGCCGGGACCTGACTGACTACCTC-3′; reverse—5′ AAACAAATAAAGCCATGCCAATCTCA-3′ with the ratio to other primers 1 : 10). Full-length SGK1 was amplified using the following primers: 5′-TTTGAGCGCTAACGTCTTTCTGT-3′ and 5′-TTGCTAAGCTTCCAGAGATGTGC-3′. SGK1 cDNA was purified from agarose gels and sequenced (Veritas, Rockville, MD, USA).

### 2.5. Ingenuity Pathways Analysis

Ingenuity Pathways Analysis (IPA) (Ingenuity Systems, Redwood City, CA, USA) was used for the identification of SGK1/FOXO3A-associated molecular paths of p53 versus ANXA7 in LNCaP. The p53-signature genes (210) from Comparative Marker Selection (GenePattern) (data not shown) were mapped to corresponding genes/proteins in IPA's database. IPA yielded the p53-centered interactome network that was analyzed for significant alterations in major cancer-related cell survival pathways with a threshold of *P* < 0.01. The two identified canonical pathways, PI3K/Akt and PTEN signaling, were first enriched with p53, ANXA7, FOXO3A, and SGK1 relations from the IPA database. Next, the PI3K/Akt and PTEN pathways were modified using Path Designer, IPA, and were further customized by adding ANXA7 versus p53 connections (genes as nodes and relationships as edges) from the current report.

## 3. Results

### 3.1. WT-ANXA7 Eliminated Androgen-Sensitive LNCaP Prostate Cancer Cells and Inhibited Cell Cycle More Effectively Than the Canonical Tumor Suppressor p53

**Figure 1:**
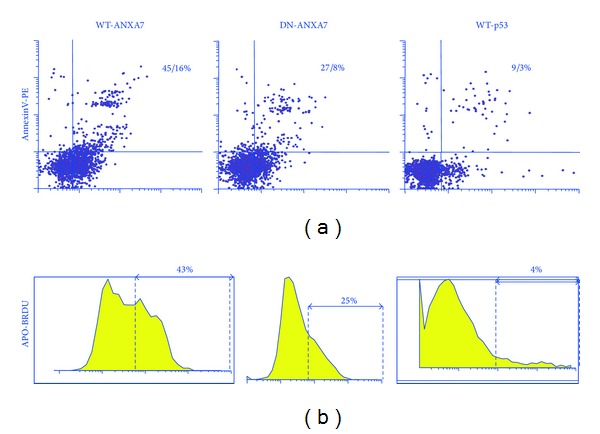
The WT/DN-ANXA7- and p53-induced cell death responses in LNCaP. PCD rates (18 h, AnnexinV-PE (a) and APO-BRDU (b)) were estimated in LNCaP cells transfected with vector alone, WT- or DN-ANXA7, and p53. Images represent typical results (%) from triplicate experiments.

**Figure 2:**
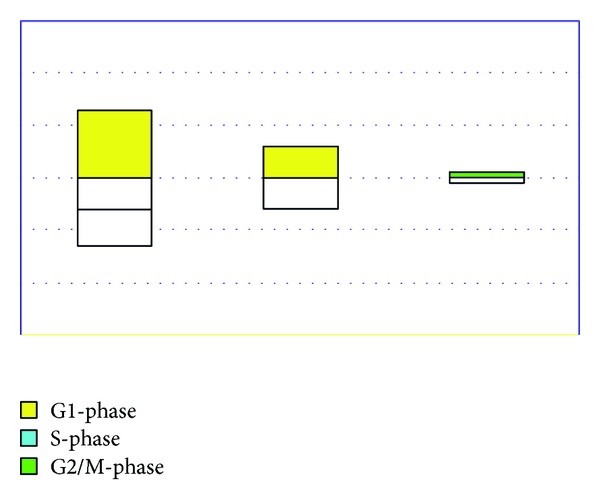
Graphs represent the differences (delta %) in cell numbers in different phases in response to WT/DN-ANXA7 and p53 (18 hr); mean values from replicates (%) are presented after the subtraction of control levels in corresponding vectors.

Programmed cell death (PCD) responses to WT/DN-ANXA7 and p53 (18 hr) were assessed by Annexin V-PE and APO-BRDU. In AnnexinV-PE assay (Figure 1(a)), WT-ANXA7 induced early apoptosis with phosphatidylserine exposure as well as late apoptosis with membrane permeabilization, whereas DN-ANXA7 failed to reach the same PCD rates (*P* < 0.001 for both comparisons) in LNCaP cells. In APO-BRDU assay (Figure 1(b)), WT-ANXA7 caused a 2-fold PCD increase compared to DN-ANXA7 (*P* < 0.001) inducing DNA fragmentation almost in half of LNCaP cells. In the meantime, p53 caused only a slight increase with the phosphatidylserine exposure causing a potentially reversible early apoptosis. In contrast, WT-ANXA7 and p53 induced comparable levels of PCD and cell growth inhibition in androgen-resistant DU145 and PC3 [4]. As shown in Figure 2, WT-ANXA7 caused a G1-arrest in LNCaP cells that was not matched by DN-ANXA7 (*P* < 0.001), whereas p53 failed to reach the cell growth inhibition effects of WT- as well as DN-ANXA7. These results along with DNA fragmentation (APO-BRDU) as well as by phosphatidylserine exposure and membrane permeabilization (AnnexinV-PE) suggest that wild-type- (WT-) ANXA7 surpassed the p53-induced PCD and cell cycle inhibition in androgen-sensitive LNCaP.

### 3.2. P53 Hyperphosphorylates FOXO3A and Reduces LMW SGK1 Products While ANXA7 Reduces FOXO3A Phosphorylation and Increases LMW SGK1

**Figure 3:**
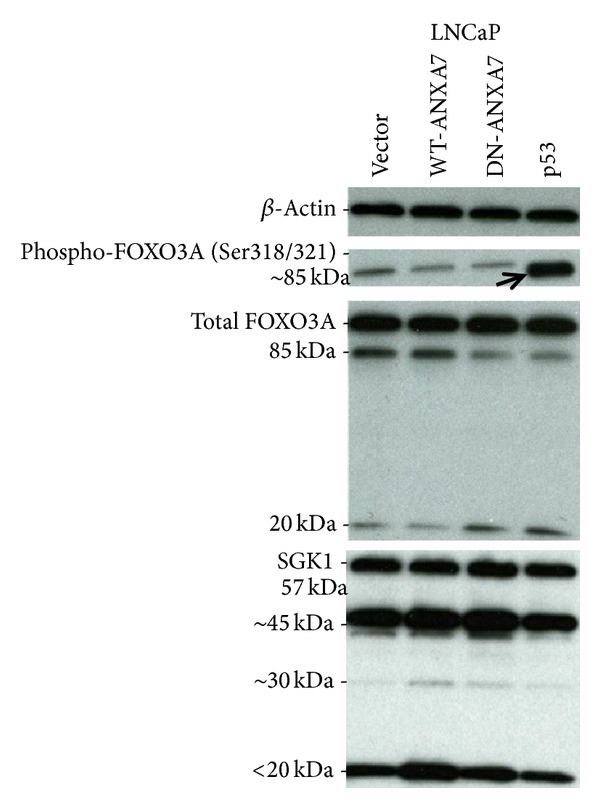
LNCaP cells transfected with vector, WT/DN-ANXA7, or p53. Protein synexpression was assessed by Western blotting. Arrows designate FOXO3A-S318/321 hyperphosphorylation in the p53-transfected LNCaP.

While similar tumor suppressor effects of WT-ANXA7 and p53 in DU145 were accompanied by similar RB-E2F profiles, the lack of RB1 dephosphorylation and* E2F* induction by p53 compared to WT-ANXA7 contributed to the p53 insufficiency in androgen-sensitive LNCaP [4]. In the PTEN-mutant LNCaP with a constitutively active Akt which is expected to phosphorylate FOXO3A [5], p53 activation can further hyperphosphorylate FOXO3A [6], thus preventing the proapoptotic FOXO3A transcription. Hence, we compared WT/DN-ANXA7 and p53 effects on the expression of FOXO3A in LNCaP. As shown in Figure 3, FOXO3A phosphorylation in LNCaP was distinctly affected by WT/DN-ANXA7 and p53 at the Ser318/321 site. Consistent with the lack of tumor suppressor effects, p53 drastically hyperphosphorylated FOXO3A-Ser318/321 in LNCaP, but not in other prostate cells (data not shown). In addition, p53 and DN- (but not WT-) ANXA7 enhanced total FOXO3A degradation: 85 kDa was reduced and 20 kDa increased. In the meantime, the expression of full-length SGK1 was not changed, while LMW SGK1 products (including aberrant <45 kDa) were enhanced by WT-ANXA7 and reduced by p53. These results suggest that the LMW-SGK1 enhancement by ANXA7 in LNCaP was accompanied by the reduced FOXO3A-S318/321 phosphorylation which indicated a nuclear translocation of FOXO3A. In contrast, the p53-induced FOXO3A-S318/321 hyperphosphorylation indicated the enhancement of FOXO3A cytoplasmic retention and deactivation in LNCaP.

### 3.3. The p53-Regulated SGK1 Kinase Was Aberrantly Transcribed and Translated in LNCaP Prostate Cancer Cells

**Figure 4:**
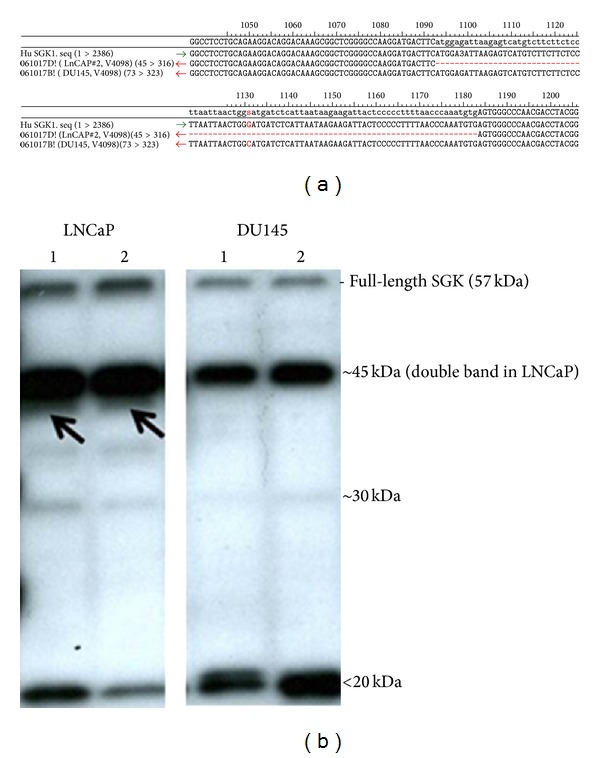
Aberrant SGK1 isoforms in LNCaP compared to DU145. (a) SGK1 cDNA from LNCaP and DU145 was sequenced and aligned with wild-type SGK1 reference. The alignment image shows the missing exon11 in LNCaP (red dotted line) and the G/C-change in DU145. (b) SGK1 isoform in LNCaP is designated by an arrow. Both cells are represented by controls only (parental and vector alone as 1 and 2, resp.).

In order to further explore a possible role of aberrant SGK1 in FOXO3A hyperphosphorylation in LNCaP, we examined SGK1 cDNA in LNCaP versus DU145. Unlike DU145, LNCaP displayed a double-band for full-length* SGK1* cDNA, thereby validating a slightly smaller size of the extra-*SGK1*-transcript (not shown). cDNA sequence analysis verified that the* SGK1 *sequences from LNCaP lacked the entire exon11 (90nt). In addition,* SGK1 *from DU145 had a G/C-change (∗) in the same exon as compared to wild-type SGK1 reference (Figure 4(a)). Distinct SGK1 protein expression profiles in parental LNCaP and DU145 maintained their cell specificity in the ANXA7- and p53-transfected cells. LNCaP possessed a smaller extra-LMW-SGK1 protein form (marked by an arrow, Figure 4(b)) that was not found in DU145 cells. Most remarkably, both ANXA7s enhanced, while p53 abolished, the extra-45kDa-SGK1. In addition, WT-ANXA7 particularly induced *∼*15 kDa-SGK1 in LNCaP. In DU145, p53 reduced the LMW-SGK1-forms, while both ANXA7s did not cause detectable changes. These results suggest that the diverse LMW-SGK1 profiles in response to ANXA7 and p53 could distinctly affect the phosphorylation of SGK1-targets.

### 3.4. ANXA7 versus p53 in AKT/SGK and FOXO3A Associated Cell Survival Signaling (Ingenuity Pathways Analysis)

**Figure 5:**
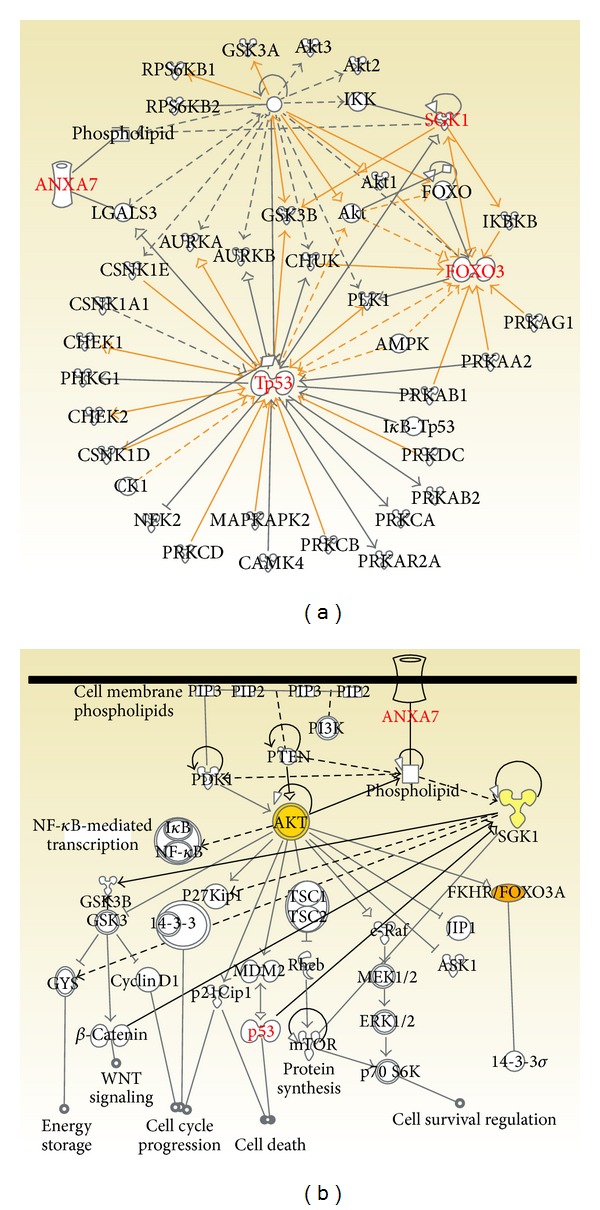
Both images were created using IPA. The left image shows ANXA7, p53, and PTEN connections to the network that involves FOXO3A and putative FOXO3A-phosphorylating kinases; the phosphorylation involving relationships are highlighted (yellow edges). The right image shows a schematic pathway that was created using PI3K/Akt canonical pathway as a template and was enriched by putative ANXA7 and SGK1 connections; FOXO3A and the FOXO3A-phosphorylating SGK1 and Akt are highlighted in yellow.

A majority of putative FOXO3A-targeting kinases could be directly connected to p53 through various experimentally proven relations including phosphorylation. Particularly, the Akt-associated signaling could provide a clue for ANXA7 success versus p53 failure in LNCaP. Acting in concert with Akt, SGK1 could propagate PI3K survival that involves exporting FOXO3A from the nucleus and inhibiting transcription of the genes that promote apoptosis and cell growth arrest. Using Ingenuity Pathway Analysis and p53 regulated genes, we positioned the SGK1/FOXO3A-cascade in the overlapping Akt pathway. Furthermore, we elucidated the role of distinct SGK1/FOXO3A-associated regulation in p53 versus ANXA7 responses and proposed that aberrant SGK1 could affect reciprocal SGK1-FOXO3A-Akt regulation (Figure 5). While ANXA7 appeared to overcome PTEN-mutant status in LNCaP, the lack of dephosphorylation by this lipid phosphatase could further fuel the p53-induced FOXO3A phosphorylation.

## 4. Discussion

This study reveals the differential role of SGK1/FOXO3A-associated molecular mechanisms in contrasting TSG effects of ANXA7 and p53 in LNCaP, a model for the androgen-sensitive prostate cancer. The novel aberrant SGK1 form that was identified in this study appeared to modulate the FOXO3A phosphorylation and, subsequently, the p53 and ANXA7 tumor suppressor effects. Although known SGK1 protein isoforms differ in their N-terminals (SwissProt), almost all predicted SGK1 splice isoforms lack the C-terminal exon11 (ASAP II, data not shown). Compared to the longer splice variants overexpressed in tumors [14], the exon11-lacking aberrant SGK1 in LNCaP is likely to affect the SGK1 kinase function versus the N-terminal-associated subcellular localization. SGK1 has been hitherto postulated to enhance cell survival. However, the aberrant SGK1 was distinctly regulated by the p53 and WT-ANXA7 with opposite cell survival effects, thereby suggesting a more complex modulation of cell survival by SGK1 splice variants. Cell-specific insufficiency of p53 in LNCAP coincided with the presence of aberrant SGK1 transcript and protein form that could potentiate baseline FOXO3A phosphorylation at the SGK1-specific site (Ser315), thereby priming the 315-318-321 site for the catalyzed phosphorylation by other kinases such as Akt. Additionally, a loss of the Ser422-neighboring exon11 may particularly affect the SGK1 recruitment in Akt/mTOR signaling. Playing a critical role in SGK1 activation, SGK1 catalytic domain is phosphorylated by PDK1 at Thr256, while Ser422 phosphorylation is catalyzed by mTORC2 which phosphorylates a similar site in Akt/PKB [15].

The opposing effects of WT- and DN-ANXA7 (the latter inhibits calcium-dependent phospholipid binding and aggregation) specifically underlined the role of annexin properties in the cell survival regulation by ANXA7. The lipid phosphatase PTEN and inositol-1,4,5-triphosphate receptor ITPR3 were reduced in our tumorigenesis-prone* Anxa7*-deficient mice that have defective nutrient response [1, 16]. On the contrary, the upregulation of PTEN by WT- (but not DN-) ANXA7 possibly restored the nutrient-sensing cell survival control in PI3K/Akt/mTOR-cascade including the serum-regulated SGK1. DN-ANXA7 (which lacks phospholipid-binding properties) failed to match the WT-ANXA7-induced PCD and G1-arrest, thereby emphasizing the importance of phospholipid-associated signaling in the LNCaP cells with mutant PTEN status and activated Akt prosurvival.

## 5. Conclusions

In conclusion, unlike p53 that was incapacitated in LNCaP, the Ca/phospholipid-binding WT-ANXA7 revived the PTEN/FOXO3A-associated cell survival control in these PTEN-deficient and androgen-sensitive prostate cancer cells. For the first time, we demonstrated that LNCaP possess aberrant* SGK1 *transcript that lacks exon11. Consistent with that, SGK1 protein profile included the extra-low-molecular-weight-SGK1-form that was abolished by p53. The initially high SGK1-specific S318/321 phosphorylation of FOXO3A was further enhanced by p53 in LNCaP, but not in DU145, implied a cytoplasmic retention. In contrast, WT-ANXA7 dephosphorylated FOXO3A in the PTEN-deficient LNCaP. The SGK1-antagonists that block androgen effects on LNCaP growth are considered prostate cancer therapeutics [17]. While phospho-FOXO3A can be an adverse prognostic factor in cancer [18], the FOXO3A transactivation is employed by anticancer drugs [19]. We propose that aberrant SGK1 could affect reciprocal SGK1-FOXO3A-Akt regulation and, hence, further studies of ANXA7 TSG effects may lead to the compelling therapeutic modulation of SGK1/FOXO3A-mediated cell survival in cancer cells.
